# Correlation of DEPDC5 rs1012068 and rs5998152 Polymorphisms with Risk of Hepatocellular Carcinoma: A Systematic Review and Meta-Analysis

**DOI:** 10.1155/2023/5957481

**Published:** 2023-01-24

**Authors:** Shaoliang Zhu, Zhenyong Tang, Mengjie Zou, Tingting Tan, Yi Tang, Yuanyuan Chen, Bin Liang, Dongyi Xie, Yongyu Yang, Shaowei Xie, Guangyuan Xie, Xiaofeng Dong, Tianqi Liu, Yuntian Tang, Jianrong Yang

**Affiliations:** ^1^Department of Hepatobiliary, Pancreas and Spleen Surgery, The People's Hospital of Guangxi Zhuang Autonomous Region, Guangxi Academy of Medical Sciences, Nanning 530021, China; ^2^Department of Gastrointestinal Endoscopy Center, The People's Hospital of Guangxi Zhuang Autonomous Region, Guangxi Academy of Medical Sciences, Nanning 530021, China; ^3^Department of Hepatobiliary, Pancreas and Spleen Surgery, Jiangbin Hospital of Guangxi Zhuang Autonomous Region, Nanning 530021, China

## Abstract

**Background:**

Emerging evidence has shown that two common genetic polymorphisms within the pleckstrin domain-containing protein 5 (DEPDC5), rs1012068 and rs5998152, may be associated with the risk of hepatocellular carcinoma (HCC), especially in those individuals chronically infected with the hepatitis C virus (HCV) or the hepatitis B virus (HBV). However, these findings have not been consistently replicated in the literature due to limited sample sizes or different etiologies of HCC. Thus, the present systematic review and meta-analysis were performed to resolve this inconsistency.

**Methods:**

The databases PubMed, Embase, Web of Science, the China National Knowledge Infrastructure, and Scopus were searched up to December 12, 2022. Data from relevant studies were pooled, and odds ratios and 95% confidence intervals were calculated.

**Results:**

A total of 11 case-control studies encompassing 2,609 cases and 8,171 controls on rs1012068 and three encompassing 411 cases and 1,448 controls on rs5998152 were included. Results indicated that the DEPDC5 rs1012068 polymorphism did not significantly increase HCC risk in the total population (allelic model (OR = 1.32, 95% CI = 1.04–1.67, *P* = 0.02); the recessive model (OR = 1.42, 95% CI = 0.96–2.10, *P* = 0.08); the dominant model (OR = 1.43, 95% CI = 1.09–1.87, *P* = 0.01); the homozygous model (OR = 1.61, 95% CI = 1.01–2.57, *P* = 0.05); the heterozygous model (OR = 1.39, 95% CI = 1.09–1.79, *P* = 0.009)). Subgroup analyses based on ethnicity and etiology revealed that the rs1012068 polymorphism, under all five genetic models, was associated with increased HCC risk in Asians or in individuals with chronic HBV infection but not in individuals with chronic HCV infection. A significant association was also observed between rs5998152 and HCV-related HCC risk in Asians chronically infected with HCV under allelic, dominant, and heterozygous models.

**Conclusion:**

Our study suggests that the DEPDC5 rs1012068 polymorphism increases HCC risk, especially in Asians with chronic HBV infection, while the rs5998152 polymorphism increases HCC risk in Asians with chronic HCV infection.

## 1. Introduction

Liver cancer is the fifth most common cancer and the fourth leading cause of cancer-related death worldwide. Among men, it is the fourth most frequent cancer and the second leading cause of cancer-related deaths [1]. Hepatocellular carcinoma (HCC) accounts for 75%–85% of cases of primary liver cancer worldwide [2]. The main risk factors for HCC are chronic infection with the hepatitis B virus (HBV) or hepatitis C virus (HCV), aflatoxin-contaminated foods, heavy alcohol intake, excess body weight, type 2 diabetes, and smoking. Besides these etiological factors, increasing evidence has revealed that host genetic variations, including single-nucleotide polymorphisms (SNPs), might also play a role in HCC development and progression.

Pleckstrin domain-containing protein 5 (DEPDC5) has been implicated in focal epilepsy, brain malformation, and sudden unexplained death in epilepsy [3–5]. DEPDC5 may be a target to treat epilepsy because it negatively regulates amino acid sensing through the signaling pathway involving the mammalian target of rapamycin complex 1 (mTORC1) [6, 7]. DEPDC5 also negatively regulates the AKT-mTORC1 pathway, so its agonists may be useful against the activation of latent HIV-1 infection [8]. DEPDC5 may participate in a signaling pathway in which Pim1 and Akt act via mTORC1 to promote the proliferation and survival of cancer cells [9]. Downregulation of DEPDC5 leads to upregulation of matrix metalloprotease 2 through the *β*-catenin pathway, which may contribute to HCV-related fibrosis [10]. Such downregulation also renders HCC tumors more resistant to reactive oxygen species under the leucine-depleted conditions of chronic liver disease, contributing to poor patient outcomes [11].

In addition to these associations between DEPDC5 and various diseases, polymorphisms in the DEPDC5 gene have been linked to the risk of HCC [12–23]. A genome-wide association study first demonstrated that the DEPDC5 variant rs1012068 could increase HCC risk in individuals with chronic HCV infection [12], and this relationship was replicated in several studies [15, 18, 20]. On the other hand, several studies did not find such a relationship [9, 14, 18]. Similarly, some studies found a significant association between rs1012068 and the risk of HBV-related HCC [13, 16], while another study failed to detect this relationship [14].

These contradictory results may reflect the relatively small samples in individual studies, heterogeneity among control populations, and different HCC etiologies. We conducted the present systematic review and meta-analysis to clarify the relationship of DEPDC5 polymorphisms rs1012068 and rs5998152 with HCC risk. We also performed subgroup analyses based on ethnicity and the etiology of HCC.

## 2. Materials and Methods

### 2.1. Search Strategy

This meta-analysis complied with “Preferred Reporting Items for Systematic Reviews and Meta-Analyses” (PRISMA) guidelines [24]. A comprehensive search for relevant studies was performed in the PubMed, Embase, Web of Science, Chinese National Knowledge Infrastructure, and Scopus databases from their inception through December 12, 2022. The following terms were used: “genetic polymorphism” or “single-nucleotide polymorphism” or “polymorphism” or “SNP” or “mutation” or “variation” or “variant,” or “liver tumor” or “liver cancer” or “hepatocellular carcinoma” or “liver neoplasms,” and “DEP domain containing 5” or “DEPDC5” or “rs1012068” or “rs5998152.” There were no language restrictions. Additional studies were identified through manual searching of references in original or review articles on this topic. If there was a duplication of published literature by the same research group, the study with the larger sample was selected. Any disagreements were resolved by discussion.

### 2.2. Inclusion and Exclusion Criteria

#### 2.2.1. Inclusion Criteria

- The study cohorts included DEPDC5 rs1012068 and rs5998152 polymorphisms in patients with HCC
- Histological features were assessed by liver biopsy, and diagnostic criteria were clearly stated
- Unrelated case-control studies were included
- If two (or more) studies included the same cohort, only the most recent was included
- Sufficient data for estimating odds ratios (ORs) and 95% confidence intervals (CIs) on the HCC risk were reported or could be calculated

#### 2.2.2. Exclusion Criteria

- The source of cases was unclear
- No clear diagnostic criteria for HCC were described
- The study was a duplicate publication
- The study was a review, meta-analysis, comment, or conference abstract
- Genotyping data were not reported in sufficient detail

### 2.3. Data Extraction

The data from the included studies were extracted by two independent investigators. Discrepancies during data extraction were resolved by a third investigator. The extracted information included the first author's surname, publication year, country in which the study was conducted, ethnicity, cohort characteristics of the cases and controls, the total number of patients in the case and control groups, the number of subjects with each genotype, and matched parameters between cases and controls.

### 2.4. Assessment of Methodological Quality

Quality assessments of the eligible studies were performed using the Newcastle–Ottawa Scale (NOS) [25]. The NOS involves a total of 9 items, each of which has a score that ranges from 1 to 9. A NOS score of 5 points or above would be classified as a high-quality study, while a NOS score of 4 points or below would be classified as a poor-quality study [26].

### 2.5. Statistical Analysis

The unadjusted odds ratio (OR) and 95% confidence interval (CI) were used to assess the correlation of DEPDC5 rs1012068 and rs5998152 polymorphisms with the risk of HCC based on the genotype frequencies in cases and controls. The Z test was used to evaluate the significance of the association, with *P* < 0.05 considered significant. When *P* > 0.10 for the Q test, meta-analysis was performed using a fixed-effect model, indicating the absence of heterogeneity among studies; otherwise, a random-effect model was used. Review Manager 5.3 (Cochrane Collaboration) was used for all statistical tests for meta-analyses. Begg's funnel plot and Egger's linear regression in Stata 12.0 software (Stata Corp., College Station, TX, USA) were used to evaluate publication bias, with *P* < 0.05 considered significant.

## 3. Results

### 3.1. Characteristics of Primary Studies

**Figure 1:**
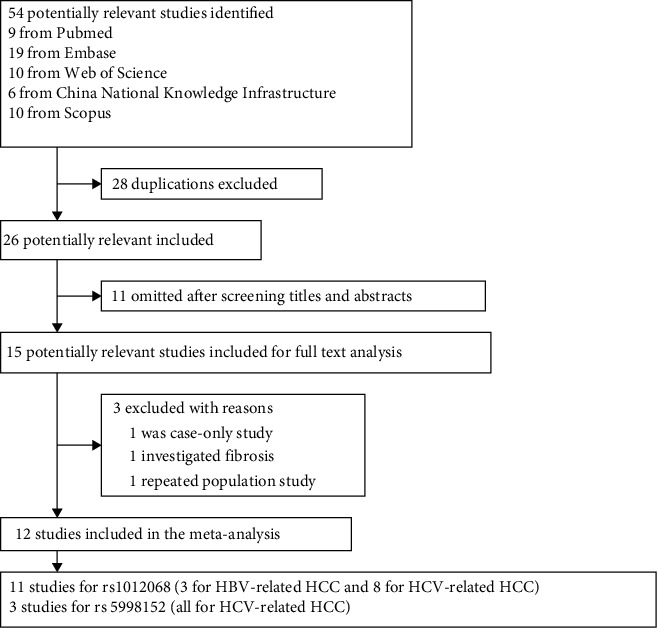
Flowchart of study selection.

The flowchart of study selection is summarized in Figure 1, and search strategies for each database are presented in Table S1. After a comprehensive search of the databases using the search strategies in Table S1, 54 relevant studies were compliant with the search strategy, of which 28 were excluded due to being duplicates. Another 11 were omitted after screening titles and abstracts. Among the 15 studies remaining, one was a case-only study [27], one investigated fibrosis but not HCC [10], and two were based on the same participants [19, 28]. Eventually, 12 studies were included in the current meta-analysis (Table 1). No relevant case-control studies were identified based on the alternative polymorphism IDs for rs1012068 (rs56511012, rs58339834, rs386510025) or for rs5998152 (rs61578881, rs8143107).

A total of 11 studies [12–22] investigated rs1012068, and 3 studies [12, 15, 23] investigated rs5998152. The distribution of genotypes in controls was consistent with Hardy-Weinberg equilibrium (HWE). The average NOS score of the 12 case-control studies was 7.09 points (ranging from 6 to 8 points), which suggested that the methodological quality of the 12 studies was generally adequate.

### 3.2. Quantitative Data Synthesis

#### 3.2.1. rs1012068 and HCC Risk

As shown in Table 2 and Figure S1, a meta-analysis based on a population of 2,609 cases and 8,171 in 11 studies [12–22] revealed that the rs1012068 polymorphism did not significantly increase HCC risk in total under the allelic model (OR = 1.32, 95% CI = 1.04–1.67, *P* = 0.02); the recessive model (OR = 1.42, 95% CI = 0.96–2.10, *P* = 0.08); the dominant model (OR = 1.43, 95% CI = 1.09–1.87, *P* = 0.01); the homozygous model (OR = 1.61, 95% CI = 1.01–2.57, *P* = 0.05); or the heterozygous model (OR = 1.39, 95% CI = 1.09–1.79, *P* = 0.009).

**Figure 2:**
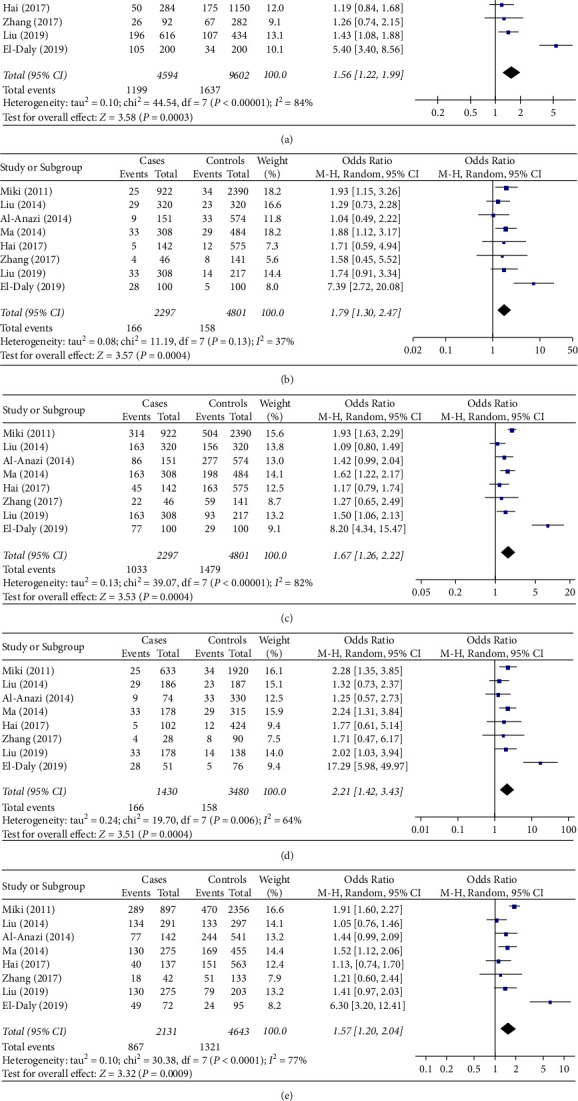
Forest plot showing the relationship between DEPDC5 rs1012068 polymorphism and HCC risk in Asians under different genetic models: (a) allelic (G vs. T), (b) recessive (GG vs. TG + TT), (c) dominant (GG + TG vs. TT), (d) homozygous (GG vs. TT), and (e) heterozygous (TG vs. TT). Abbreviations: DEPDC5, pleckstrin domain-containing protein 5; HCC, hepatocellular carcinoma; CI, confidence interval; d*f*, degree of freedom; M-H, Mantel-Haenszel.

A meta-analysis based on ethnicity for the subgroup of 2,297 Asian cases and 4,801 Asian controls in 8 studies [12, 14–20] showed that the rs1012068 polymorphism significantly increased HCC risk in Asians (Table 2; Figure 2) under the allelic model (OR = 1.56, 95% CI = 1.22–1.99, *P* < 0.001); the recessive model (OR = 1.82, 95% CI = 1.43–2.30, *P* < 0.001); the dominant model (OR = 1.67, 95% CI = 1.26–2.22, *P* = 0.004); the homozygous model (OR = 2.21, 95% CI = 1.42–3.43, *P* < 0.001); and the heterozygous model (OR = 1.57, 95% CI = 1.20–2.04, *P* < 0.001). Subgroup analysis in Caucasian populations was not performed because only two studies reported such data.

**Figure 3:**
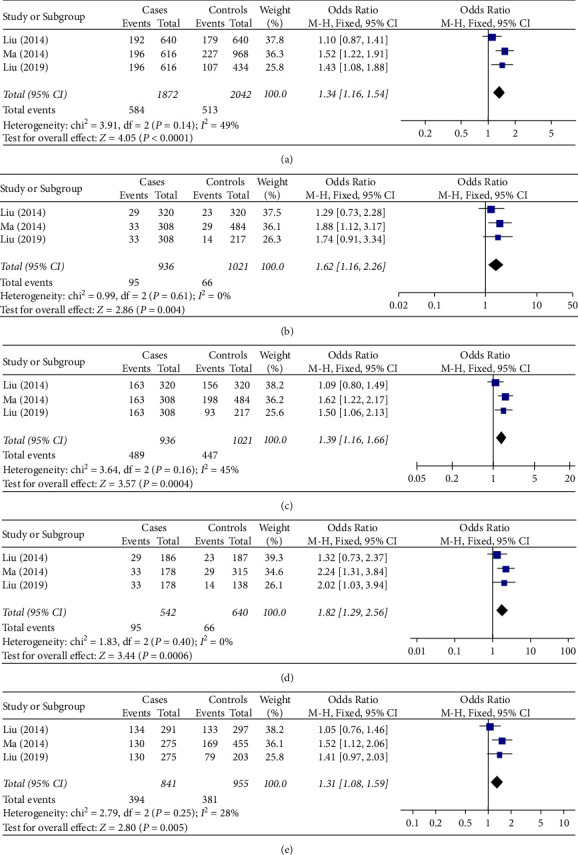
Forest plot showing the relationship between DEPDC5 rs1012068 polymorphism and HCC risk in individuals with chronic HBV infection under different genetic models: (a) allelic (G vs. T), (b) recessive (GG vs. TG + TT), (c) dominant (GG + TG vs. TT), (d) homozygous (GG vs. TT), and (e) heterozygous (TG vs. TT). Abbreviations: DEPDC5, pleckstrin domain-containing protein 5; HBV, hepatitis B virus; HCC, hepatocellular carcinoma; CI, confidence interval; d*f*, degree of freedom; M-H, Mantel-Haenszel.

Then, we conducted a meta-analysis based on the etiology of HCC, in which both cases and controls were chronically infected with HBV. Results for the subgroup of 936 cases and 1,021 controls in 3 studies [14, 16, 19] showed that the rs1012068 polymorphism significantly increased HCC risk in individuals with chronic HBV infection (Table 2; Figure 3) under the allelic model (OR = 1.34, 95% CI = 1.16–1.54, *P* < 0.001); the recessive model (OR = 1.62, 95% CI = 1.16–2.26, *P* = 0.004); the dominant model (OR = 1.39, 95% CI = 1.16–1.66, *P* < 0.001); the homozygous model (OR = 1.82, 95% CI = 1.29–2.56, *P* < 0.001); and the heterozygous model (OR = 1.31, 95% CI = 1.08–1.59, *P* = 0.005).

Next, a meta-analysis was conducted based on the etiology of HCC, in which both cases and controls were chronically infected with HCV. Results for the subgroup of 1,673 cases and 7,150 controls in 8 studies [12, 13, 15, 17, 18, 20–22] showed that the rs1012068 polymorphism did not significantly increase HCC risk in individuals with chronic HCV infection (Table 2; Figure S2) under the allelic model (OR = 1.46, 95% CI = 1.03–2.05, *P* = 0.03); the recessive model (OR = 1.63, 95% CI = 1.00–2.66, *P* = 0.05); the dominant model (OR = 1.56, 95% CI = 1.04–2.34, *P* = 0.03); the homozygous model (OR = 1.91, 95% CI = 0.99–3.65, *P* = 0.05); and the heterozygous model (OR = 1.48, 95% CI = 1.02–2.16, *P* = 0.04).

#### 3.2.2. rs5998152 and HCC Risk

**Figure 4:**
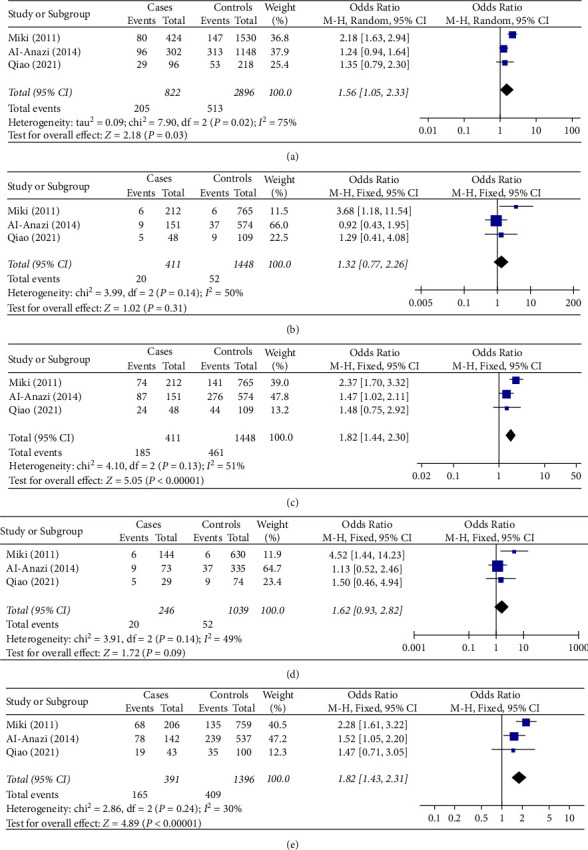
Forest plot showing the relationship between the DEPDC5 rs5998152 polymorphism and HCC risk in Asians with chronic HBV infection under different genetic models: (a) allelic (T vs. C), (b) recessive (TT vs. CT + CC), (c) dominant (CT + TT vs. CC), (d) homozygous (TT vs. CC), and (e) heterozygous (CT vs. CC). Abbreviations: DEPDC5, pleckstrin domain-containing protein 5; HBV, hepatitis B virus; HCC, hepatocellular carcinoma; CI, confidence interval; d*f*, degree of freedom; M-H, Mantel-Haenszel.

As shown in Table 2 and Figure 4, a meta-analysis based on a population of 411 cases and 1,448 controls in 3 studies [12, 15, 23] revealed that the rs5998152 polymorphism was significantly associated with HCC risk in Asians with chronic HCV infection under the allelic model (OR = 1.56, 95% CI = 1.05–2.33, *P* = 0.03); the dominant model (OR = 1.82, 95% CI = 1.44–2.30, *P* < 0.001); and the heterozygous model (OR = 1.82, 95% CI = 1.43–2.31, *P* < 0.001); but not under the recessive model (OR = 1.32, 95% CI = 0.77–2.26, *P* = 0.31); or the homozygous dominant model (OR = 1.62, 95% CI = 0.93–2.82, *P* = 0.09).

### 3.3. Sensitivity Analysis

The controls in all 8 case-control studies that investigated the association between the rs1012068 polymorphism and HCC risk were chronically infected with HCV, except the controls in one study [20], in which the controls were healthy individuals. To eliminate such heterogeneity among controls, we repeated the meta-analysis after deleting this study. Repeating the meta-analysis led to similar results as when the study was included, suggesting that our meta-analysis is reliable (Figure S3).

### 3.4. Publication Bias

**Figure 5:**
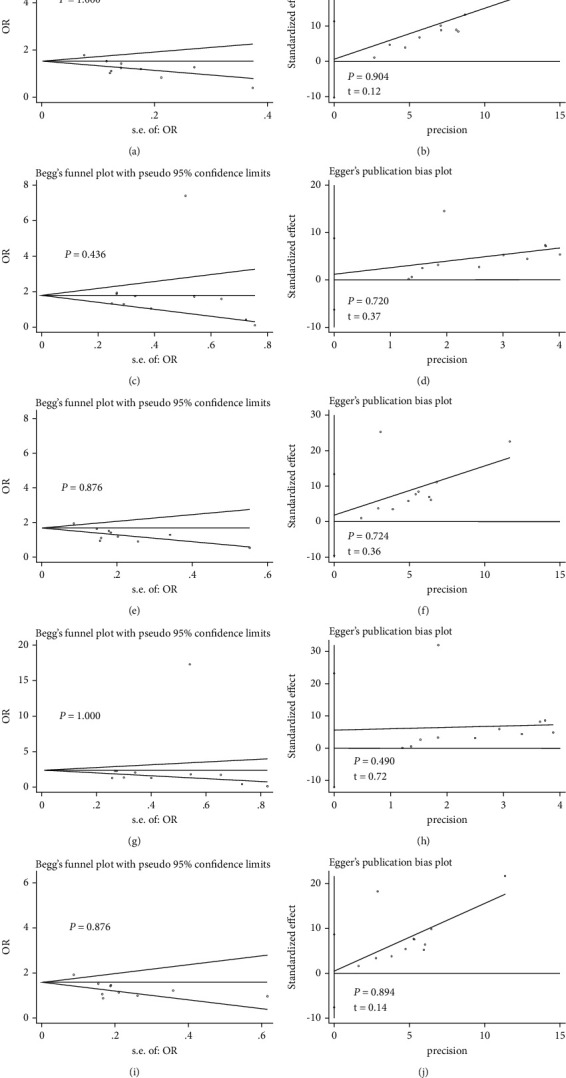
Begg's funnel plot to assess publication bias in the meta-analysis of the association between the DEPDC5 rs1012068 polymorphism and HCC risk in the total population under different genetic models: (a) allelic (G vs. T), (c) recessive (GG vs. TG + TT), (e) dominant (GG + TG vs. TT), (g) homozygous (GG vs. TT), and (i) heterozygous (TG vs. TT). Egger's regression test to assess publication bias in the meta-analysis of the association between DEPDC5 rs1012068 polymorphism and HCC risk in the total population under different genetic models: (b) allelic (G vs. T), (d) recessive (GG vs. TG + TT), (f) dominant (GG + TG vs. TT), (h) homozygous (GG vs. TT), and (j) heterozygous (TG vs. TT). Abbreviations: DEPDC5, pleckstrin domain-containing protein 5; HCC, hepatocellular carcinoma; OR, odds ratio.

**Figure 6:**
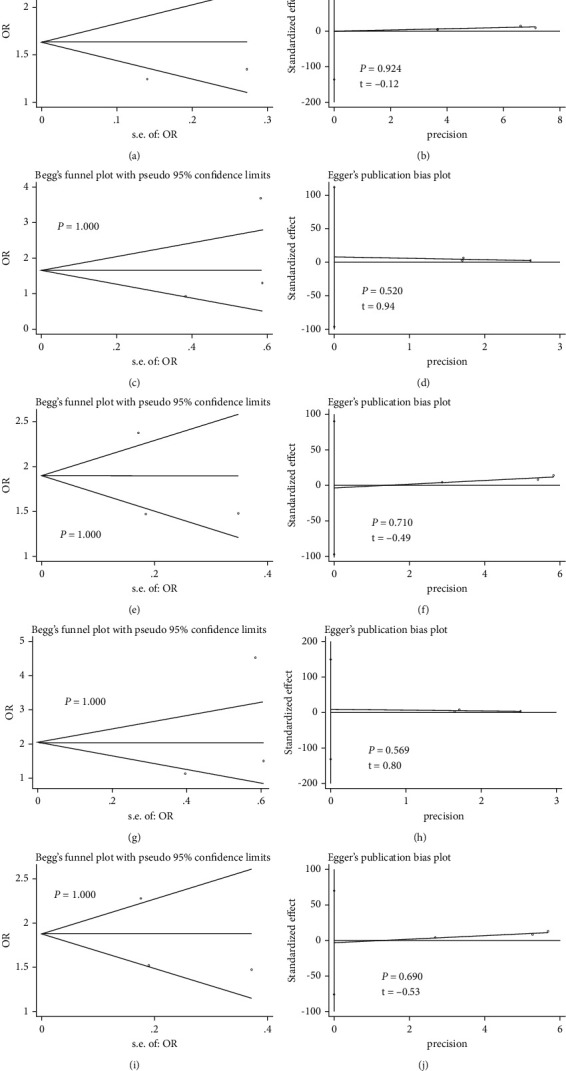
Begg's funnel plot to assess publication bias in the meta-analysis of the association between DEPDC5 rs5998152 polymorphism and HCC risk in the total population under different genetic models: (a) allelic (C vs. T), (c) recessive (CC vs. TC + TT), (e) dominant (CC + TC vs. TT), (g) homozygous (CC vs. TT), and (i) heterozygous (TC vs. TT). Egger's regression test to assess publication bias in the meta-analysis of the association between DEPDC5 rs5998152 polymorphism and HCC risk in the total population under different genetic models: (b) allelic (C vs. T), (d) recessive (CC vs. TC + TT), (f) dominant (CC + TC vs. TT), (h) homozygous (CC vs. TT), and (j) heterozygous (TC vs. TT). Abbreviations**:** DEPDC5, pleckstrin domain-containing protein 5; HCC, hepatocellular carcinoma; OR, odds ratio.

As shown in Figures 5 and 6, Begg's funnel plot and Egger's regression test showed that the meta-analysis of rs1012068 and rs5998152 polymorphisms showed no obvious asymmetry under the five genetic models (all *P* > 0.05).

## 4. Discussion

In the case of rs1012068, an overall meta-analysis of the total population indicated a significant association with increased HCC risk, regardless of HCC etiology and source of controls. Subgroup analysis based on ethnicity supported this association for Asians. Subsequently, meta-analyses of individuals chronically infected with HBV or HCV were performed. The cases and controls in three case-control studies [14, 16, 19] were all chronically infected with HBV, and in this uniform sample, results showed that the rs1012068 polymorphism significantly increased HCC risk in individuals with chronic HBV infection. In contrast, the association between the rs1012068 polymorphism and HCV-related HCC risk was not significant.

In the case of rs5998152, three case-control studies examined a potential relationship between this polymorphism and the risk of HCV-related HCC [12, 15, 23]. All cases and controls were chronically infected with HCV. Results showed the rs5998152 polymorphism was significantly associated with HCC risk in Asians with chronic HCV infection under allelic, dominant, and heterozygous models.

It may be that these polymorphisms weaken the activity of DEPDC5, preventing it from inhibiting mTORC1 as it does normally, which in turn leads to pathogenic inflammation and cell growth in the liver [22, 29]. Future research should explore how the rs1012068 and rs5998152 polymorphisms affect DEPDC5 expression and activity.

Although positive results were obtained, some limitations that may affect the interpretation of the meta-analysis were presented in this work. First, samples were relatively small due to the lack of case-control studies, especially for rs5998152. Second, among studies investigating the association between the rs1012068 polymorphism and HCC risk, the controls in all case-control studies except one [20] were chronically infected with HCV. When one study with healthy controls was deleted from the meta-analysis [20], the results were not substantially altered, suggesting that our meta-analysis is reliable. Third, the included studies in our meta-analysis spanned 2011–2022, during which antiviral treatments have improved and been widely used for treating HCV- or HBV-related liver disease [30, 31]. Since the included studies did not report detailed data on the use of such therapies, further research should explore how they influence the risk of HCC in individuals with DEPDC5 polymorphisms. Fourth, the robustness of the current meta-analysis may be reduced because the case-control studies involved used different genotyping methods that may differ in sensitivity and specificity, and potentially by other confounding factors such as age, sex, alcohol intake, and tumor status. Given these various limitations, the findings of our meta-analysis should be validated and extended in large, well-designed studies.

In summary, our study suggests that the DEPDC5 rs1012068 polymorphism increases HCC risk, especially in Asians with chronic HBV infection, while the rs5998152 polymorphism increases HCC risk in Asians with HCV infection. Further large, well-designed studies are required to validate these findings.

## Figures and Tables

**Table 1:** Characteristics of the included studies and genotype distributions.

Study (year of publication)	Country	Ethnicity	*n* (cases), *n* (controls)	Cohort characteristics	Genotype			Allele		NOS score	Control source	Genotyping method	*P*for HWE	Matched parameters
*rs1012068*					*TT*	*TG*	*GG*	*T*	*G*					
Miki et al. [12]	Japan	Asian	922	HCV-related HCC	608	289	25	1505	339	8		Human610-quad		Age, sex, BMI
			2390	Chronic HCV infection	1886	470	34	4242	538		HB		0.446	
Lange et al. [13]	Switzerland	Caucasian	64	HCV-related HCC	36	26	2	98	30	7		Allele-specific PCR		Sex
			1849	With chronic hepatitis C	987	727	135	2701	997		HB		0.943	
Liu [14]	China	Asian	320	HBV-related HCC	157	134	29	448	192	8		PCR-RFLP		Age, sex
			320	With chronic hepatitis B	164	133	23	461	179		HB		0.573	
Al-Anazi et al. [15]	Saudi Arabia	Asian	151	HCV-related HCC	65	77	9	207	95	7		INNO-LiPA HCV II		BMI
			574	With chronic hepatitis C	297	244	33	838	310		HB		0.061	
Ma et al. [16]	China	Asian	308	HBV-related HCC	145	130	33	420	196	6		MALDI-TOF MS		-
			484	With chronic hepatitis B	286	169	29	741	227		HB		0.546	
Hai et al. [17]	Japan	Asian	142	HCV-related HCC	97	40	5	234	50	6		TaqMan		-
			575	With chronic hepatitis C	412	151	12	975	175		HB		0.671	
Zhang [18]	China	Asian	46	HCV-related HCC	24	18	4	66	26	8		MALDI-TOF MS		Age, BMI
			141	With chronic hepatitis C	82	51	8	215	67		HB		0.985	
Liu et al. [19]	China	Asian	308	HBV-related HCC	145	130	33	420	196	6		MALDI-TOF MS		-
			217	With chronic hepatitis B	124	79	14	327	107		HB		0.767	
El-Daly et al. [20]	Saudi Arabia	Asian	100	HCV-related HCC	23	49	28	95	105	8		TaqMan		Age, sex
			100	Healthy control	71	24	5	166	34		HB		0.135	
Sharkawy et al. [21]	Australia	Caucasian	188	With chronic hepatitis C	102	65	21	269	107	8		TaqMan		Age, sex, BMI
			1501	HCV-related HCC	791	580	130	2162	840		HB		0.110	
Hanan et al. [22]	Egypt	African	60	With chronic hepatitis C	27	30	3	84	36	6		TaqMan		-
			20	HCV-related HCC	6	7	7	19	21		HB		0.182	

*rs5998152*					*TT*	*TC*	*CC*	*T*	*C*					
Miki et al. [12]	Japan	Asian	212	HCV-related HCC	138	68	6	344	80	8		Human610-quad		Age, sex, BMI
			765	With chronic hepatitis C	624	135	6	1383	147		HB		0.658	
Al-Anazi et al. [15]	Saudi Arabia	Asian	151	HCV-related HCC	64	78	9	206	96	7		INNO-LiPA HCV II		BMI
			574	With chronic hepatitis C	298	239	37	835	313		HB		0.233	
Qiao et al. [23]	China	Asian	48	HCV-related HCC	24	19	5	67	29	8		MALDI-TOF MS		Age, sex, BMI
			109	With chronic hepatitis C	65	35	9	165	53		HB		0.183	

Abbreviations: HB, hospital-based; HCV, hepatitis C virus; HBV, hepatitis B virus; HWE, Hardy-Weinberg equilibrium; NOS, Newcastle–Ottawa Scale; BMI, body mass index; -, not reported.

**Table 2:** Overall meta-analysis of the association of the DEPDC5 rs1012068 and rs5998152 with hepatocellular carcinoma risk.

Genetic models		Number of studies (references)	OR (95% CI)	Z (*P* value)	d*f* (*P* value)	*I* ^2^ (%)	Meta-analysis model
*rs1012068*							
Allelic model (G vs. T)	Overall	11 [9–19]	1.32 (1.04, 1.67)	2.26 (0.02)	10 (<0.001)	86	Random
	Asians	8 [10–17]	1.56 (1.22, 1.99)	3.58 (<0.001)	7 (<0.001)	84	Random
	HBV-related	3 [11, 13, 16]	1.34 (1.16, 1.54)	4.05 (<0.001)	2 (0.14)	49	Fixed
	HCV-related	8 [9, 10, 12, 14, 15, 17–19]	1.29 (0.91, 1.84)	1.43 (0.15)	7 (<0.001)	90	Random
Recessive model (GG vs. TG + TT)	Overall	11 [9–19]	1.42 (0.96, 2.10)	1.73 (0.08)	10 (0.001)	66	Random
	Asians	8 [10–17]	1.82 (1.43, 2.30)	4.94 (<0.001)	7 (0.13)	37	Fixed
	HBV-related	3 [11, 13, 16]	1.62 (1.16, 2.26)	2.86 (0.004)	2 (0.61)	0	Fixed
	HCV-related	8 [9, 10, 12, 14, 15, 17–19]	1.26 (0.68, 2.32)	0.73 (0.47)	7 (<0.001)	75	Random
Dominant model (GG + TG vs.TT)	Overall	11 [9–19]	1.43 (1.09, 1.87)	2.58 (0.01)	10 (<0.001)	83	Random
	Asian	8 [10–17]	1.67 (1.26, 2.22)	3.53 (0.004)	7 (<0.001)	82	Random
	HBV-related	3 [11, 13, 16]	1.39(1.16, 1.66)	3.57 (<0.001)	2 (0.16)	45	Fixed
	HCV-related	8 [9, 10, 12, 14, 15, 17–19]	1.44 (0.97, 2.14)	1.82 (0.07)	7 (<0.001)	87	Random
Homozygous model (GG vs. TT)	Overall	11 [9–19]	1.61 (1.01, 2.57)	1.98 (0.05)	10 (<0.001)	75	Random
	Asians	8 [10–17]	2.21 (1.42, 3.43)	3.51 (<0.001)	7 (0.006)	64	Random
	HBV-related	3 [11, 13, 16]	1.82 (1.29, 2.56)	3.44 (<0.001)	2 (0.40)	0	Fixed
	HCV-related	8 [9, 10, 12, 14, 15, 17–19]	1.44 (0.70, 2.99)	0.99 (0.32)	7 (<0.001)	81	Random
Heterozygous model (TG vs. TT)	Overall	11 [9–19]	1.39 (1.09, 1.79)	2.62 (0.009)	10 (<0.001)	78	Random
	Asians	8 [10–17]	1.57 (1.20, 2.04)	3.32 (<0.001)	7 (<0.001)	77	Random
	HBV-related	3 [11, 13, 16]	1.31 (1.08, 1.59)	2.80 (0.005)	2 (0.25)	28	Fixed
	HCV-related	8 [9, 10, 12, 14, 15, 17–19]	1.44 (1.01, 2.07)	1.99 (0.05)	7 (<0.001)	83	Random

*rs5998152*							
Allelic model (C vs. T)	Asians/HCV-related	3 [10, 12, 20]	1.56 (1.05, 2.33)	2.18 (0.03)	2 (0.02)	75	Random
Recessive model (CC vs. TC + TT)	Asians/HCV-related	3 [10, 12, 20]	1.32 (0.77, 2.26)	1.02 (0.31)	2 (0.14)	50	Fixed
Dominant model (CC + TC vs.TT)	Asians/HCV-related	3 [10, 12, 20]	1.82 (1.44, 2.30)	5.05 (<0.001)	2 (0.13)	51	Fixed
Homozygous model (CC vs. TT)	Asians/HCV-related	3 [10, 12, 20]	1.62 (0.93, 2.82)	1.72 (0.09)	2 (0.14)	49	Fixed
Heterozygous model (TC vs. TT)	Asians/HCV-related	3 [10, 12, 20]	1.82 (1.43, 2.31)	4.89 (<0.001)	2 (0.24)	30	Fixed

Abbreviations: OR, odds ratio; 95% CI, 95% confidence interval; d*f*, degree of freedom; HBV, hepatitis B virus; HCV, hepatitis C virus.

## Data Availability

The data used to support the findings of this study are included within the article.

## Supplementary Materials

Supplementary Materials
*Figure S1.* Forest plot showing the relationship between the DEPDC5 rs1012068 polymorphism and HCC risk in the total population under different genetic models: (A) allelic (G vs. T), (B) recessive (GG vs. TG + TT), (C) dominant (GG + TG vs. TT), (D) homozygous (GG vs. TT), and (E) heterozygous (TG vs. TT). *Figure S2.* Forest plot showing the relationship between DEPDC5 rs1012068 polymorphism and HCC risk in individuals with chronic HCV infection under different genetic models: (A) allelic (G vs. T), (B) recessive (GG vs. TG + TT), (C) dominant (GG + TG vs. TT), (D) homozygous (GG vs. TT), and (E) heterozygous (TG vs. TT). *Figure S3.* Forest plot showing the relationship between the DEPDC5 rs1012068 polymorphism and HCC risk in sensitivity analysis: (A) allelic (G vs. T), (B) recessive (GG vs. TG + TT), (C) dominant (GG + TG vs. TT), (D) homozygous (GG vs. TT), and (E) heterozygous (TG vs. TT). *Table S1.* Search strategies for each database.
