# Supplementary material for: Correlation of DEPDC5 rs1012068 and rs5998152 Polymorphisms with Risk of Hepatocellular Carcinoma: A Systematic Review and Meta-Analysis
Source: J Oncol. 2023 Jan 24;2023:5957481. doi: 10.1155/2023/5957481 (PMC9889158; doi:10.1155/2023/5957481)

Figure S2

Forest plot showing the relationship between DEPDC5 rs1012068 polymorphism and HCC risk in individuals with chronic HCV infection under different genetic models: (A) allelic (G vs. T), (B) recessive (GG vs. TG + TT), (C) dominant (GG + TG vs. TT), (D) homozygous (GG vs. TT) and (E) heterozygous (TG vs. TT).

Abbreviations: DEPDC5, pleckstrin domain-containing protein 5; HCV, hepatitis C virus; HCC, hepatocellular carcinoma; CI, confidence interval; df, degree of freedom; M-H, Mantel-Haenszel.

A

Study or Subgroup	Cases Events	Cases Total	Controls Events	Controls Total	Weight	Odds Ratio M-H, Random, 95% CI
Miki (2011)	339	1844	538	4780	14.6%	1.78 [1.53, 2.06]
Lange (2013)	30	128	997	3698	12.4%	0.83 [0.55, 1.26]
Al-Anazi (2014)	95	302	310	1148	13.7%	1.24 [0.94, 1.63]
Hai (2017)	50	284	175	1150	13.1%	1.19 [0.84, 1.68]
Zhang (2017)	26	92	67	282	11.2%	1.26 [0.74, 2.15]
El-Daly (2019)	105	200	34	200	11.9%	5.40 [3.40, 8.56]
Sharkawy (2019)	107	376	840	3002	14.0%	1.02 [0.81, 1.30]
Hanan (2022)	36	120	21	40	9.1%	0.39 [0.19, 0.81]
**Total (95% CI)**		**3346**		**14300**	**100.0%**	**1.29 [0.91, 1.84]**
Total events	788		2982			

Heterogeneity: Tau² = 0.22; Chi² = 68.67, df = 7 (P < 0.00001); I² = 90%

Test for overall effect: Z = 1.43 (P = 0.15)

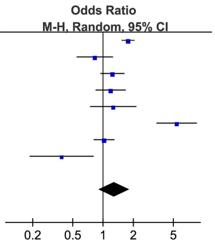

B

Study or Subgroup	Cases Events	Cases Total	Controls Events	Controls Total	Weight	Odds Ratio M-H, Random, 95% CI
Miki (2011)	25	922	34	2390	16.2%	1.93 [1.15, 3.26]
Lange (2013)	2	64	135	1849	9.3%	0.41 [0.10, 1.69]
Al-Anazi (2014)	9	151	33	574	14.4%	1.04 [0.49, 2.22]
Hai (2017)	5	142	12	575	11.9%	1.71 [0.59, 4.94]
Zhang (2017)	4	46	8	141	10.5%	1.58 [0.45, 5.52]
El-Daly (2019)	28	100	5	100	12.4%	7.39 [2.72, 20.08]
Sharkawy (2019)	21	188	130	1501	16.5%	1.33 [0.81, 2.16]
Hanan (2022)	3	60	7	20	8.9%	0.10 [0.02, 0.43]
**Total (95% CI)**		**1673**		**7150**	**100.0%**	**1.26 [0.68, 2.32]**
Total events	97		364			

Heterogeneity: Tau² = 0.54; Chi² = 28.13, df = 7 (P = 0.0002); I² = 75%

Test for overall effect: Z = 0.73 (P = 0.47)

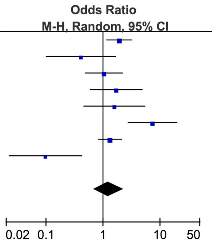

C

Study or Subgroup	Cases Events	Cases Total	Controls Events	Controls Total	Weight	Odds Ratio M-H, Random, 95% CI
Miki (2011)	314	922	504	2390	15.5%	1.93 [1.63, 2.29]
Lange (2013)	28	64	862	1849	12.7%	0.89 [0.54, 1.47]
Al-Anazi (2014)	86	151	277	574	14.1%	1.42 [0.99, 2.04]
Hai (2017)	45	142	163	575	13.7%	1.17 [0.79, 1.74]
Zhang (2017)	22	46	59	141	10.9%	1.27 [0.65, 2.49]
El-Daly (2019)	77	100	29	100	11.3%	8.20 [4.34, 15.47]
Sharkawy (2019)	86	188	710	1501	14.6%	0.94 [0.69, 1.27]
Hanan (2022)	33	60	14	20	7.3%	0.52 [0.18, 1.55]
**Total (95% CI)**		**1673**		**7150**	**100.0%**	**1.44 [0.97, 2.14]**
Total events	691		2618			

Heterogeneity: Tau² = 0.26; Chi² = 54.41, df = 7 (P < 0.00001); I² = 87%

Test for overall effect: Z = 1.82 (P = 0.07)

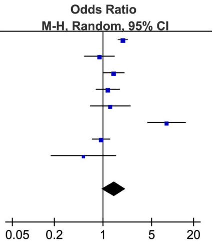

D

Study or Subgroup	Cases Events	Cases Total	Controls Events	Controls Total	Weight	Odds Ratio M-H, Random, 95% CI
Miki (2011)	25	633	34	1920	15.4%	2.28 [1.35, 3.85]
Lange (2013)	2	38	135	1122	10.1%	0.41 [0.10, 1.71]
Al-Anazi (2014)	9	74	33	330	14.0%	1.25 [0.57, 2.73]
Hai (2017)	5	102	12	424	12.3%	1.77 [0.61, 5.14]
Zhang (2017)	4	28	8	90	11.0%	1.71 [0.47, 6.17]
El-Daly (2019)	28	51	5	76	12.4%	17.29 [5.98, 49.97]
Sharkawy (2019)	21	123	130	921	15.5%	1.25 [0.76, 2.08]
Hanan (2022)	3	30	7	13	9.2%	0.10 [0.02, 0.48]
**Total (95% CI)**		**1079**		**4896**	**100.0%**	**1.44 [0.70, 2.99]**
Total events	97		364			

Heterogeneity: Tau² = 0.82; Chi² = 37.57, df = 7 (P < 0.00001); I² = 81%

Test for overall effect: Z = 0.99 (P = 0.32)

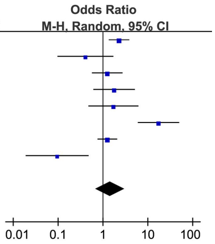

E

Study or Subgroup	Cases Events	Cases Total	Controls Events	Controls Total	Weight	Odds Ratio M-H, Random, 95% CI
Miki (2011)	289	897	470	2356	16.6%	1.91 [1.60, 2.27]
Lange (2013)	26	62	727	1714	12.8%	0.98 [0.59, 1.64]
Al-Anazi (2014)	77	142	244	541	14.6%	1.44 [0.99, 2.09]
Hai (2017)	40	137	151	563	14.0%	1.13 [0.74, 1.70]
Zhang (2017)	18	42	51	133	10.4%	1.21 [0.60, 2.44]
El-Daly (2019)	49	72	24	95	10.7%	6.30 [3.20, 12.41]
Sharkawy (2019)	65	167	580	1371	15.1%	0.87 [0.63, 1.21]
Hanan (2022)	30	57	7	13	5.9%	0.95 [0.28, 3.19]
**Total (95% CI)**		**1576**		**6786**	**100.0%**	**1.44 [1.01, 2.07]**
Total events	594		2254			

Heterogeneity: Tau² = 0.20; Chi² = 40.50, df = 7 (P < 0.00001); I² = 83%

Test for overall effect: Z = 1.99 (P = 0.05)

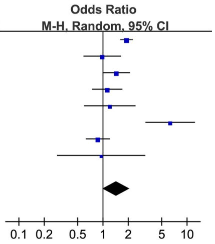

Supplement: Supplementary Materials — Figure S1. Forest plot showing the relationship between the DEPDC5 rs1012068 polymorphism and HCC risk in the total population under different genetic models: (A) allelic (G vs. T), (B) recessive (GG vs. TG + TT), (C) dominant (GG + TG vs. TT), (D) homozygous (GG vs. TT), and (E) heterozygous (TG vs. TT). Figure S2. Forest plot showing the relationship between DEPDC5 rs1012068 polymorphism and HCC risk in individuals with chronic HCV infection under different genetic models: (A) allelic (G vs. T), (B) recessive (GG vs. TG + TT), (C) dominant (GG + TG vs. TT), (D) homozygous (GG vs. TT), and (E) heterozygous (TG vs. TT). Figure S3. Forest plot showing the relationship between the DEPDC5 rs1012068 polymorphism and HCC risk in sensitivity analysis: (A) allelic (G vs. T), (B) recessive (GG vs. TG + TT), (C) dominant (GG + TG vs. TT), (D) homozygous (GG vs. TT), and (E) heterozygous (TG vs. TT). Table S1. Search strategies for each database. [file 5957481.f1.zip › Figure S2 (2).pdf]
