# Supplementary material for: Correlation of DEPDC5 rs1012068 and rs5998152 Polymorphisms with Risk of Hepatocellular Carcinoma: A Systematic Review and Meta-Analysis
Source: J Oncol. 2023 Jan 24;2023:5957481. doi: 10.1155/2023/5957481 (PMC9889158; doi:10.1155/2023/5957481)
Supplement: Supplementary Materials — Figure S1. Forest plot showing the relationship between the DEPDC5 rs1012068 polymorphism and HCC risk in the total population under different genetic models: (A) allelic (G vs. T), (B) recessive (GG vs. TG + TT), (C) dominant (GG + TG vs. TT), (D) homozygous (GG vs. TT), and (E) heterozygous (TG vs. TT). Figure S2. Forest plot showing the relationship between DEPDC5 rs1012068 polymorphism and HCC risk in individuals with chronic HCV infection under different genetic models: (A) allelic (G vs. T), (B) recessive (GG vs. TG + TT), (C) dominant (GG + TG vs. TT), (D) homozygous (GG vs. TT), and (E) heterozygous (TG vs. TT). Figure S3. Forest plot showing the relationship between the DEPDC5 rs1012068 polymorphism and HCC risk in sensitivity analysis: (A) allelic (G vs. T), (B) recessive (GG vs. TG + TT), (C) dominant (GG + TG vs. TT), (D) homozygous (GG vs. TT), and (E) heterozygous (TG vs. TT). Table S1. Search strategies for each database. [file 5957481.f1.zip › Table S1 (1).pdf]

Table S1. Search strategies for each database.

Database	Potential relevant records	Records after duplicates removed	Records after screening titles and abstracts	Records excluded with reasons	Records included in final meta-analysis
PubMed	n = 9	n = 9	n = 9	n = 2	n = 7
Embase	n = 19	n = 10	n = 0	n = 0	n = 0
Web of Science	n = 10	n = 0	n = 0	n = 0	n = 0
CNKI	n = 6	n = 6	n = 5	n = 1	n = 4
Scopus	n = 10	n = 1	n = 1	n = 0	n =1

Abbreviations: CNKI, Chinese National Knowledge Infrastructure; HCV, hepatitis C virus; HBV, hepatitis B virus.
